# Genetically Modified Lactic Acid Bacteria: a Promising Mucosal Delivery Vector for Vaccines

**DOI:** 10.1007/s12602-025-10667-3

**Published:** 2025-07-31

**Authors:** Md. Rayhan Chowdhury, Ariful Islam, Valentina Yurina, Takeshi Shimosato

**Affiliations:** 1 Graduate School of Medicine, Science and Technology, Shinshu University, Minamiminowa, Nagano, 399-4598 Japan; 2 Department of Genetic Engineering and Biotechnology, University of Rajshahi, Rajshahi, 6205 Bangladesh; 3 Department of Pharmacy, Faculty of Medicine, Universitas Brawijaya, Malang, 65145 Indonesia; 4 Institute for Biomedical Sciences, Shinshu University, Minamiminowa, Nagano, Kamiina 399-4598 Japan; 5 Institute for Aqua Regeneration, Shinshu University, Minamiminowa, Nagano, Kamiina 399-4598 Japan

**Keywords:** GmLAB, Mucosal vaccines, Vectors, Immune system, Vaccine

## Abstract

The advent of mucosal vaccines that target the primary entry points of many pathogens has revolutionized the field of immunology. Genetically modified lactic acid bacteria (gmLAB), which include genera such as *Lactobacillus* and *Bifidobacterium*, have emerged as promising vectors for delivering antigens to mucosal surfaces. These gram-positive, non-pathogenic microorganisms exhibit inherent probiotic properties, can survive through the gastrointestinal tract, and efficiently interact with the host immune system. Advances in genetic engineering have enabled the expression of a wide range of antigens in gmLAB that promote systemic and mucosal immunity. Studies have demonstrated that gmLAB-based mucosal vaccines can elicit both mucosal and systemic immune responses, providing protective immunity against specific pathogenic infections. In addition, gmLAB vectors offer good safety profiles, stability, and cost-effective production compared to traditional vaccine platforms. Recent studies demonstrated the potential of LAB vaccines in preventing infections caused by viral, bacterial, and parasitic pathogens and in immunotherapy for treating allergies and cancers. This review highlights the mechanisms underlying gmLAB-based mucosal vaccine delivery, current advancements, challenges, and prospects in recombinant mucosal vaccines.

## Introduction

Vaccines are a cornerstone of public health medicine and confer immunity against a variety of infectious organisms and viruses, thereby preventing millions of deaths annually [1]. Vaccination is crucial for protecting children, especially those under 5 years of age. Six standard vaccines DTP; measles, mumps, and rubella (MMR); polio; and hepatitis B save over 5 million lives annually [2]. Although these vaccines are capable of preventing over a million fatalities annually, more than 1.4 million children under the age of 5 years nevertheless die each year from these infectious diseases, predominantly in underdeveloped nations [2]. The primary cause of these fatalities is inadequate vaccine coverage and distribution. An effective strategy to address these inadequacies and prevent fatalities would be to develop vaccines that elicit a robust immune response against infectious agents while also being readily accessible and economically viable [3]. However, challenges in developing countries, including high costs, rapidly increasing populations, and high disease burdens, make it essential to develop more cost-effective vaccines.

Live bacterial vaccines that include probiotic lactic acid bacteria (LAB) have been studied for their immunomodulatory actions and potential as novel vaccine adjuvants [4]. These bacteria mimic natural infections and can be administered either orally or nasally, thus ensuring greater acceptance and safety [5]. Genetically modified LAB (gmLAB), which are modified using recombinant DNA technology, hold significant promise as vectors for mucosal vaccines due to their ability to induce robust immune responses [6]. Mucosal vaccines play a crucial role in preventing infectious diseases by inducing local mucosal immunity, which can provide protection against pathogens. Although the development of mucosa-targeting vaccines has been limited to date by a variety of factors, efforts are ongoing to overcome these challenges and improve vaccine efficacy [7].

Intranasal vaccination has emerged as a promising strategy for inducing mucosal immunity against respiratory pathogens such as influenza [8] and coronavirus 2 (SARS-CoV-2) [9]. While genetically modified lactic acid bacteria (gmLAB) have already demonstrated protective efficacy against influenza [10], recent studies suggest that they may also hold potential for combating SARS-CoV-2 via intranasal administration [11].

Recent research has underscored the necessity of developing mucosal vaccine technology further to address infectious disease outbreaks [12]. Antigen-specific secretory immunoglobulin A (sIgA) responses at mucosal sites and efficacious systemic immune responses can be induced through mucosal delivery of vaccines [13]. Additionally, mucosal immunization is convenient for vaccination, associated with good compliance, and eliminates the risk of blood-borne illness transmission through contaminated syringes because the vaccines do not require professional medical personnel for inoculation and contain extremely low endotoxin levels compared with injected vaccines, due to strict quality control practices [14, 15]. Vaccines are also available for mucosal immunization, which offers advantages over traditional injection-based vaccination methods. Research into the use of gmLAB as live vectors for mucosal vaccination has demonstrated their potential for application in the delivery of DNA vaccines and antigens for immune prophylaxis [16]. Due to their favorable safety profile, LAB have been widely utilized in food production and preservation applications, and as probiotics. These genetically modified organisms have been used to successfully deliver functional proteins for treating a variety of pathologies, making them a cost-effective option for treatment [17]. gmLAB may also play a role in advancing the development of next-generation DNA vaccine platforms and delivery systems [18].

Recent technological advances in genomics and structural biology have transformed vaccine development and enabled the generation of mRNA platforms that have greatly accelerated the development of new and improved vaccines [19]. These mRNA vaccine platforms represent a revolutionary approach to immunization by facilitating the delivery of synthetic mRNA sequences that encode specific viral antigens. mRNA-based vaccines stimulate the immune system to recognize and mount robust humoral and cellular immune responses to the target pathogen [20]. Advantages of mRNA vaccine platforms include rapid development and production, non-infectiousness, strong immunogenicity, and adaptability. However, challenges include stability and storage, as mRNA molecules require ultra-cold storage conditions and the use of lipid nanoparticles for effective delivery. Despite these challenges, mRNA vaccines consistently demonstrate efficacy in head-to-head comparisons with viral vector vaccines, inducing higher levels of specific antibodies [21]. In addition to mRNA vaccines, DNA-based vaccines have also been developed because they offer several advantages over traditional antigen-based vaccines**.** DNA vaccines are cost-effective, simple to manufacture, and stable, and they induce strong and long-lasting immune responses without a risk of infection [22]. Moreover, the ability to produce bioactive molecules using gmLAB could lead to the development of effective cancer treatments using these organisms [23, 24].

The use of gmLAB represents a cost-effective strategy for combating infections, particularly through mucosal vaccination. gmLAB vaccines can be administered mucosally via the oral or intranasal routes. Such non-invasive administration negates the need for needles and syringes, thus reducing medical waste and lowering the risks of needle-borne infections. Moreover, gmLAB-based vaccines are more amenable for mass vaccination campaigns, as they do not require specially trained healthcare personnel for administration, which further lowers operational costs [25]. gmLAB can be produced via scalable, cost-effective fermentation methods commonly used in the food industry, ensuring high reproducibility. They can be lyophilized and remain stable at room temperature or under standard refrigeration, reducing cold chain dependence. Their potential for self-administration also facilitates mass distribution with minimal infrastructure. In contrast, mRNA vaccines generally require complex manufacturing and ultra-cold storage, posing logistical challenges.

The aim of this review is to provide a comprehensive analysis of gmLAB as potential vectors for mucosal vaccines. We will explore the mechanisms of gmLAB-based vaccine delivery, evaluate current advancements, and address the challenges associated with their development. Additionally, the review will discuss the future prospects of gmLAB as a transformative platform for mucosal immunization and vaccine applications.

## Characteristics of gmLAB

LAB are gram-positive, nonsporulating bacteria that can ferment carbohydrates into lactic acid. The group comprises bacilli and cocci from various genera, including *Lactococcus*, *Lactobacillus*, *Streptococcus*, *Pediococcus*, *Leuconostoc*, and *Bifidobacterium*. The Food and Drug Administration has granted “generally regarded as safe” (GRAS) status to numerous LAB; however, this classification also encompasses pathogenic bacteria such as *Streptococcus pyogenes* and *Streptococcus pneumoniae* [26]. Because LAB lack genes encoding proteins involved in several biosynthesis pathways, they are often present in environments rich in amino acids, purines, and pyrimidines [27]. These bacteria have been used in food production and preservation applications, and some species are recognized as probiotics, which the World Health Organization defines as organisms that offer health benefits to the host when administered in sufficient quantities [28]. Probiotics include LAB strains that maintain intestinal microbiota homeostasis by modulating the bacterial flora, stimulating the immune system, and suppressing allergic reactions. Probiotics are essential for gastrointestinal health, and some strains even protect against harmful bacteria by competing for colonization surfaces, inhibiting pathogen growth through compounds they produce, or stimulating mucosal epithelial cells to produce mucus and antimicrobial peptides [29].

Probiotics play a crucial role in the management of a variety of gastrointestinal problems, including inflammatory bowel disease (IBD), autoimmune disorders, and diarrhea caused by viruses or antibiotic use [30]. gmLAB have been extensively investigated for more than 20 years as possible bacterial carriers of preventative or therapeutic substances. LAB enables immunization via the mucosal route, which is more straightforward than standard injection-based methods and provides greater effectiveness against pathogens that exploit the mucosa as the primary entry point into the body. LAB strains induce both mucosal and systemic immune responses [31]. Their resistance to the low pH of gastric fluid and their capacity to adhere to intestinal epithelial surfaces are significant determinants of their attractiveness in immunoprophylaxis and therapy. Some LAB isolates exhibit adjuvant properties, which enhance the immune response induced by the antigens they carry. LAB are thus an appealing alternative to other vectors utilized in vaccine construction, as their application does not necessitate specialized personnel or low-temperature storage [16].

gmLAB are capable of surviving passage through the gastrointestinal tract, which enables them to colonize the intestine, where they play a critical immunological role [32]. Considerable recent research has focused on gram-positive bacterial vehicles, with a primary emphasis on indigenous or food bacteria, including LAB (*Lactococcus*, *Streptococcus*, and *Lactobacillus*), non-pathogenic species of *Staphylococcus* (*S. carnosus* and *S. xylosus*), and attenuated strains of *Listeria monocytogenes*, which are particularly prone to elicit an MHC class I–restricted cytotoxic immune response (Table 1) [33]. gmLAB are increasingly used as antigen delivery systems due to their amenability to genetic manipulation. The genetic characteristics of LAB have been well characterized, allowing for the development of efficient transformation protocols and precise control of antigen expression [34]. Furthermore, LAB can be transformed with recombinant DNA using techniques such as electroporation or conjugation. Various constitutive and inducible promoters have been developed for LAB, which enable balancing of high expression levels with the maintenance of bacterial cellular health (Table 2) [35]. Integration vectors can facilitate the stable incorporation of antigen-encoding genes into the LAB genome, and codon optimization can improve the efficiency of antigen expression. Genetic tools are available that enable the secretion of antigens into the extracellular environment or the display of antigens on the surface of LAB cells [36].

**Table 1:** Comparison of gmLAB, *Salmonella*, and *Listeria monocytogenes* as mucosal vaccine vectors

Vector	Antigen(s)	Expression Location	Route	Immune Outcome	Refs
*Lactococcus lactis*	Tetanus toxin fragment C	CYT	Oral	Serum IgG1/IgG2a, fecal IgA; protective efficacy comparable to intranasal	[158]
			Intranasal	Serum and mucosal antibodies; protection without colonization	[159]
	*Brucella* lumazine synthase	SEC	Oral	Anti-BLS IgG, Th1/Th2 cytokines; cellular protection	[148]
	EspB	CYT	Oral	Serum IgG, mucosal IgA, Th1, Th2, challenge	[160]
	SOD	SEC	Oral	Serum IgM and IgG (IgG2a dominated), mucosal IgA, challenge	[161]
	SEB	SEC	Oral	Serum IgG, mucosal IgA, challenge	[162]
	HPV-16 E7 + murine IL-12	SEC, CWA	Intranasal	Enhanced Th1 (IL-2, IFN-γ); IL-12 boosted mucosal/systemic protection	[163]
	HIV-1 subtype C Gag	CWA	Oral, nasal	Serum IgG, mucosal IgA, DC activation, CTL	[164]
	Rotavirus VP7	CYT, SEC, CWA	Oral	Neutralizing antibody	[165]
	Rotavirus VP8	SEC, CWA	Oral	Serum IgG, mucosal IgA, neutralizing antibodies	[166, 167]
	FAdV-4 Hexon DC-targeting peptide	CWA	Oral	Protective immunity in poultry against FAdV-4	[168]
	NP	CYT	Oral	Serum IgG, mucosal IgA, Th1, Th2, challenge (only with CTB)	[169]
	NA	CWA	Oral	Serum IgG, mucosa IgA, NI titer, challenge	[169]
	HA1	CWA	Oral	Serum IgG, mucosal IgA (only with CTB), splenocyte proliferation, cytokine production (only with CTB), challenge (only with CTB)	[170]
*Lactiplantibacillus plantarum*	NP and matrix protein DC-targeting peptide	CWA	Oral	Th1, Th2, Th17, CTL, challenge	[171]
	Influenza virus M2e antigen	CYT	Oral	DC activation, Th1, CTL, mucosal IgA, challenge	[172]
*Lactiplantibacillus casei*	*Salmonella* FliC (flagellin)	CWA	Oral	Th1 response (CD4⁺ IFN-γ⁺); protection against *S. Enteritidis* without strong humoral response	[173]
	PCV2 Cap + *E. coli* LTB tag	SEC	Oral	Systemic IgG & mucosal IgA; stronger response with pPG612.1 vector	[174]
*Salmonella typhimurium*	HIV-1 subtype C Gag	CYT	Oral	Gag-specific CD4⁺ Th1 & Th2 cytokines; serum IgG1/IgG2a	[175]
	*Helicobacter pylori* HpaA, NAP, UreA/B	SEC	Oral	Gastric IgA, systemic IgG1/2c; protection against *H. pylori* colonization	[176]
*Listeria. monocytogenes*	HIV Gag, FIV Gag/Env DNA vaccine	CYT	Oral, vaginal	Long-lived CD8⁺ and CD4⁺ T-cell responses; mucosal/systemic protection	[177]
	Influenza virus NP	CYT	Intranasal	Systemic/mucosal immunity; partial protection in mice	[178]
	HSV-1 epitope	CYT	Oral	Protective CTL response; Th1-biased	[177]

*HIV*, human immunodeficiency virus, *HA,* hemagglutinin; *NP*, influenza virus A nucleoprotein; *NA*, influenza A neuraminidase; *HA1*, influenza virus A hemagglutinin; *LTB*, heat-labile enterotoxin B subunit; *CTB*, cholera toxin B subunit; *NI*, neuraminidase inhibition; *CTL*, cytotoxic T lymphocyte; *EHEC*, enterohemorrhagic *Escherichia coli*; *SOD*, superoxide dismutase; *SEB*, staphylococcal enterotoxin type B; *NT*, neutralizing antibody; *CYT*, cytoplasmic; *SEC*, secreted; *CWA*, cell-wall anchored

**Table 2:** Genetic tools for antigen expression in LAB

Mechanism	Examples & Functionality	Refs
Constitutive promoter	Ppgm, Pldh, PslpA (*Lactobacillus*); P45, P32 (*L. lactis*)–drive continuous expression with varying intensity	[179, 180]
Inducible promoter	Nisin-NICE system (PnisA, PnisF, PnisZ); carbohydrate-responsive promoters (PFOS, Plac, Ptre); tetracycline- and IPTG-inducible systems for tunable control	[181, 182]
Integration vectors	Chromosomal insertion systems that avoid plasmid instability and ensure long-term expression without antibiotic selection	[183, 184]
Codon optimization	HPV-E7 codon redesign; BSH codon optimization; GFP expression improvement–all enhance translation efficiency	[141, 185]
Secretion/surface display	Vectors using SP-PrtP, SP-SlpA, SP-Usp45 for extracellular secretion or for anchoring antigens on the cell surface	[96, 183]

## gmLAB as Oral and Intranasal Vaccines

**Fig. 1:**
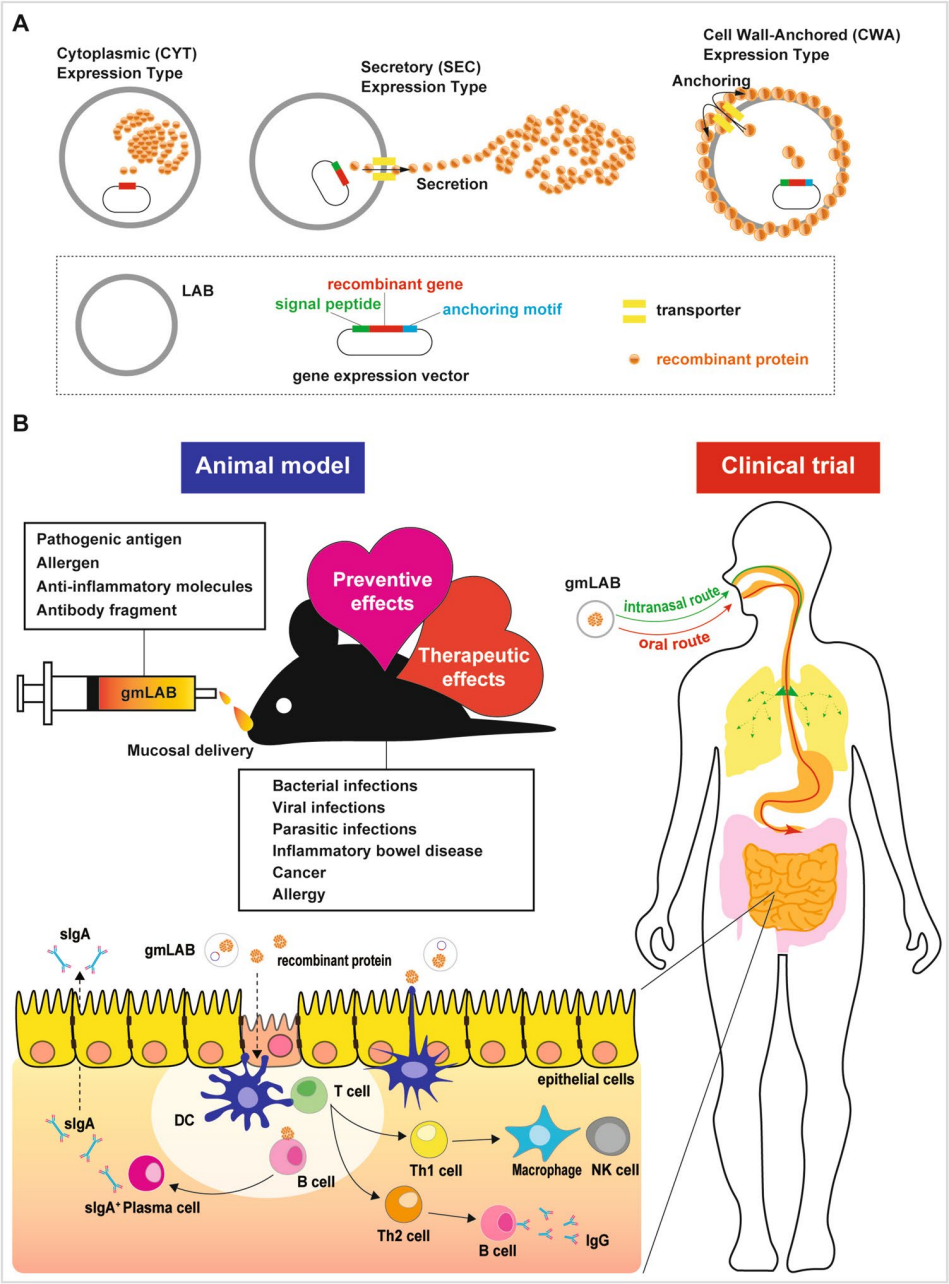
Schematic representation of recombinant protein expression patterns in gmLAB and administration routes. **A:** Three expression patterns have been reported: cytoplasmic expression type (CYT type), in which the recombinant protein is retained within the bacterial cells; the secretory expression type (SEC type), in which the recombinant protein is secreted extracellularly; and the cell wall–anchored type (CWA type), in which the recombinant protein is anchored to the bacterial cell wall. **B:** In the mucosal immune response, M cells transport antigens to dendritic cells (DCs), which migrate to the T-cell zone and prime naïve T cells. Depending on cytokine signals, T cells differentiate into subsets, such as Th1, which activates macrophages, and Th2, which promotes B cell class switching to IgG and IgA production. Activated T cells and follicular DCs in the germinal center further differentiate B cells into IgA⁺ plasma cells, which migrate to the intestinal lamina propria and secrete polymeric IgA for mucosal defense. This panel also includes conceptual illustrations of immune activation observed in animal models and potential applications in human clinical trials

gmLAB are also being investigated for potential use as oral and intranasal delivery systems due to their ability to survive in the interaction with mucosal surfaces and stimulate immune responses (Fig. 1). Because gmLAB can enhance both non-specific inflammatory responses and specific immune responses, they are positioned as potentially ideal and innovative candidates for use as live oral and intranasal vaccine carriers [37] (Fig. 1). Depending on the promoter used and the vector design, recombinant proteins expressed by gmLAB can be secreted, displayed on the cell surface, or retained intracellularly. For instance, expression systems utilizing inducible (e.g., PnisA), constitutive (e.g., P32, Pldh), or stress-inducible promoters (e.g., PgroESL) have been employed in the development of these different expression modes [17]. Existing LAB oral vaccines include *Lactococcus lactis* expressing human papilloma virus (HPV) antigens for preventing cervical cancer, *Lactobacillus casei* that produces *Helicobacter pylori* urease subunit B for preventing gastric infections, and *Lactobacillus plantarum* that carries influenza virus antigens for imparting protective immunity [31]. However, challenges associated with factors such as antigen stability, control of expression, and efficiency of mucosal delivery need to be addressed further. Recent advancements in CRISPR-Cas9 genome editing and synthetic biology techniques have significantly enhanced the precision and effectiveness of gmLAB applications. For instance, Wiull et al. developed a dual-plasmid CRISPR-Cas9 system that enabled efficient chromosomal insertion of expression cassettes into *Lactiplantibacillus plantarum* WCFS1, achieving surface display and intracellular expression of SARS-CoV-2 antigens, a notable improvement in mucosal vaccine applications [38]. Similarly, Zhou et al. demonstrated a seamless genome editing strategy in *L. plantarum* using CRISPR-Cas9–assisted recombineering, which allowed for the introduction of gene knockouts, point mutations, and insertions without introducing exogenous DNA, ultimately leading to the production of *N*-acetylglucosamine (GlcNAc), thereby showcasing the industrial application potential of this system [39]. Wu et al. reviewed the use of CRISPR-based genome editing tools in LAB and highlighted how CRISPRi (interference) can be used to downregulate gene expression without permanent genomic alteration, thereby adding flexibility in engineering probiotic functionalities [40]. Collectively, these technologies mark the beginning of a transformative phase in LAB engineering, as they enhance the stability, safety, and functional efficacy of gmLAB as live delivery vectors.

Antigens in the gastrointestinal tract are affected by the harsh environment and low pH. Numerous delivery vehicles and encapsulation strategies have been developed to protect and preserve the structural integrity of antigens and facilitate their release at sites that will induce phagocytosis by antigen-presenting cells (APCs). Both living and non-living systems have been developed [41]. The recognition of pathogen-associated molecular patterns (PAMPs) by pattern-recognition receptors (PRRs) results in the release of inflammatory cytokines and sIgA production in intestinal tissues, as evidenced by the potent adjuvant properties of living delivery vehicles, such as live bacterial strains and viral vectors [42]. A variety of recombinant bacterial strains, including *Salmonella*, *L. monocytogenes*, and LAB, have been employed as antigen delivery vehicles for oral vaccination [43]. These strains can express antigens from a variety of pathogens to induce protection against the corresponding pathogens (Table 1). Vaccines against tetanus, *H. pylori*, EHEC/ETEC, *Clostridium difficile*, *Salmonella enterica*, rotaviruses, *C. albicans*, avian influenza virus, and parasites such as *Plasmodium yoelii* and *Giardia lamblia* are available options. Nevertheless, the status of these recombinant bacterial strains as genetically modified organisms necessitates they be engineered in such a way as to ensure their survival within the host [44].

## Advantages of Using gmLAB for Vaccine Development

With regard to vaccine development and licensure, the advantages of using gmLAB include their potential as carriers for vaccine antigens to mucosal tissues, their demonstrated effectiveness in inducing antigen-specific immunity, and their ability to induce cell-mediated immunity and memory cells [45]. gmLAB have been shown to trigger immune responses in mice, and specific strains such as *L. casei* CRL 431, *L. acidophilus* CRL 924, *L. delbrueckii* subsp. *bulgaricus* CRL 423, and *Streptococcus thermophilus* CRL 412 have been shown to enhance non-specific immune responses [46]. However, further research is needed to determine the optimal strains, doses, timing of administration, and safety considerations for human use. Additionally, gmLAB have been used to modulate allergic immune responses in mice, showing effectiveness in shifting toward non-allergic immune responses to specific allergens [46, 47]. The specific mechanisms by which gmLAB stimulate immunity include activation of the systemic and secretory immune responses through complex interactions in the intestinal ecosystem and sending signals to activate immune cells (Fig. 1) [48]. gmLAB enhances non-specific immune responses by stimulating the phagocytic activity of macrophages and releasing lysosomal enzymes. However, the fate of these bacteria within tissues remains poorly understood, as they may not directly contact epithelial cells in healthy intestinal tissue [49].

Interactions between gmLAB and host immune components are primarily mediated through PRRs such as Toll-like receptors (TLRs) on epithelial cells and APCs [50]. These PRRs recognize microbial-associated molecular patterns, which in turn triggers innate immune pathways and the production of pro-inflammatory cytokines and chemokines (e.g., interleukin [IL]−6, tumor necrosis factor [TNF]-α, and interferon [IFN]-α/β) [51]. This signaling cascade ultimately recruits immune cells and primes adaptive responses. Notably, strains such as *Lactobacillus rhamnosus* and *L. delbrueckii* have been shown to activate these responses through interactions with PRRs to promote dendritic cell (DC) maturation and cytokine release [52]. These interactions are causally linked to downstream effects, such as enhanced migration of immune cells, expansion of IgA-producing B cells, and activation of CD4⁺ T cells within the intestinal lamina propria. Thus, mucosal immunity triggered by gmLAB is a consequence of initial PRR-mediated recognition rather than direct interactions with T or B cells [53]. Different LAB strains such as *L. casei*, *L. plantarum*, *L. rhamnosus*, *S. thermophilus*, *L. delbrueckii* subsp. *bulgaricus*, and *Lactococcus lactis* interact with different immune cells at the small intestine and bronchus levels, affecting IgA + cells and CD4 + T cells differently. LAB also stimulates cytokine release from epithelial cells, thereby activating T cells and inducing the production of other cytokines by T cells associated with the lamina propria.

Whether gmLAB are processed by the mucosal immune system depends on multiple factors, including the microbial environment, host characteristics, and antigen design [54]. In healthy intestinal tissues, gmLAB may not directly contact epithelial cells, which could limit their uptake and immune processing. However, in cases of inflammation or mucosal barrier disruption, APCs such as DCs and macrophages are more likely to sample these bacteria and initiate immune responses [55]. Key factors that affect the development of an immune response in these cases include the *gut microbiota composition*, which plays a regulatory role in mucosal immunity. Dysbiosis—commonly observed in individuals with inflammatory diseases or in malnourished children—has been linked to impaired processing and reduced effectiveness of mucosal vaccines [56]. Additionally, the *dose and type of expressed antigen*—whether cytoplasmic, secreted, or cell wall-anchored (CWA)-determined antigen accessibility and immune cell activation [57]. CWA forms, for instance, facilitate better recognition and uptake by M cells and DCs [58]. Host-specific variables such as immune status and *age* also play a role. Elderly individuals often exhibit immune senescence, which reduces vaccine responsiveness, whereas children may lack established mucosal immune memory, making dose and adjuvant optimization essential [59].

gmLAB can induce immune cells to produce pro-inflammatory cytokines such as IFN-α/β, IL-1β, and TNF-α. For example, *L. acidophilus* has been shown to induce the production of IFN-α/β by murine peritoneal macrophages, whereas *L. delbrueckii* subsp. *bulgaricus* and *S. thermophilus* can cause the production of IL-1β and TNF-α [60]. LAB are GRAS and widely used in food products and probiotics [61]. The natural immunostimulatory effects of LAB can enhance the efficacy of vaccines without requiring additional chemical adjuvants due to their ability to colonize mucosal surfaces, their capacity to stimulate both innate and adaptive immune responses, and their potential for low-cost production [62]. The introduction of gmLAB into the gut environment can affect the balance within the existing commensal microbiota through mechanisms such as competitive exclusion, nutrient competition, and modulation of host immune responses [63]. For instance, certain LAB strains such as *Lactobacillus plantarum* exhibit antimicrobial properties that may inhibit the growth of other beneficial strains such as *Lactococcus lactis*, thereby altering the microbiota equilibrium when used in mixed formulations [64]. Furthermore, gmLAB can compete for adhesion sites on the intestinal epithelium as well as for nutrients, which can lead to the displacement of indigenous microbes and thereby impact microbial diversity and stability [65]. The outcome of such interactions is often strain-specific and may depend on the expression system, surface antigens presented by the gmLAB, as well as metabolic products such as lactic acid and bacteriocins [66].

## Targeting Respiratory Pathogens

gmLAB have been developed that express antigens from various respiratory pathogens, and vaccines using these LAB have shown effectiveness in inducing protective immunity against pathogens such as *Streptococcus pneumoniae* [67]. As LAB strains can enhance the immune response against infections, their use in prevention strategies is considered promising [68]. The effectiveness of gmLAB in vaccines against respiratory pathogens has been attributed to the expression of pathogen antigens that induce protective immunity at the site of pathogen entry. For instance, intranasal administration of heat-killed *Lactobacillus casei* DK128 in mice was shown to confer protection against several subtypes of influenza A virus, including H1N1 and H3N2. This protection was attributed to enhanced innate and adaptive immune responses, such as increased alveolar macrophage activity and virus-specific antibody production [69].

Bacterium-like particles derived from *Lactococcus lactis* have also been utilized as nasal vaccine carriers. These non-viable particles display antigens on the surface, leading to the induction of robust mucosal and systemic immune responses. BLPs have been explored for use in vaccines against a variety of respiratory pathogens, leveraging their safety and efficacy in inducing protective immunity [70]. Furthermore, recombinant *Lactobacillus casei* expressing pneumococcal surface antigen A has been shown to reduce nasal colonization of *Streptococcus pneumoniae* in mice, highlighting the potential for the use of gmLAB in preventing bacterial respiratory infections [71]. The survival of strains during production, packaging, and storage must also be tested and declared [72]. The combination of *L. casei*, *L. acidophilus*, *L. salivarius*, and *Lc. lactis* showed the highest efficacy in an in vivo study. *Lactobacillus plantarum* inhibited all previously mentioned LAB strains except *Lactococcus lactis*, which reduced the in vivo efficacy of the four-strain mixture [73].

## Preventing Gastrointestinal Diseases

Mucosal vaccines delivered via gmLAB have demonstrated particular effectiveness in preventing gastrointestinal diseases due to their ability to survive in the harsh gastrointestinal environment and induce both systemic and mucosal immune responses [74]. Various strategies have been employed to overcome the challenges presented by the gastrointestinal tract’s low pH, enzymatic degradation, and mucosal barrier function, including encapsulation and the use of protective delivery vehicles [75]. Encapsulation methods involving polymers such as alginate or chitosan preserve antigen integrity, thereby ensuring effective delivery and enhanced uptake by mucosal-associated lymphoid tissue [76]. In addition, recombinant gmLAB strains of *Lactococcus lactis* and *Lactobacillus plantarum* have been engineered to express and deliver antigens specific to gastrointestinal pathogens to elicit effective protective mucosal immunity [77].

Antigen delivery using live bacterial vectors enhances recognition through PAMPs, which engage PRRs to stimulate cytokine release and the production of sIgA, which is crucial for protection against gastrointestinal infections [42, 78]. For instance, recombinant *L. lactis* strains expressing antigens from *Helicobacter pylori*, *Clostridium difficile*, and rotavirus have demonstrated effectiveness in animal models, highlighting delivery via the oral route as an advantageous strategy due to its simplicity, safety, and ability to induce mucosal immunity [79–81]. Rotavirus infection is the most common cause of infantile diarrhea and leads to a significant number of deaths, especially in underdeveloped nations [82]. Probiotic bacteria have been shown to provide health benefits in terms of protection against rotavirus infection, as infants administered *Lactobacillus reuteri* recovered 1 day earlier and exhibited enhanced specific immunoglobulin responses compared with control infants [83]. Administration of *L. casei* strains to children with acute diarrhea was also shown to trigger an immune response against rotaviruses. Several studies clearly showed that some probiotic strains are effective for treating acute rotavirus diarrhea in children [82, 84]. Integrating these oral vaccine strategies within the broader context of gastrointestinal disease prevention showcases the potential of gmLAB to serve as mucosal vaccine platforms capable of effectively targeting a variety of pathogenic organisms.

LAB have also been used for the prevention and treatment of gastrointestinal inflammatory disorders such as IBD and mucositis and showed promise in animal models prior to clinical trials in humans [45]. IBDs such as Crohn’s disease and ulcerative colitis are chronic conditions characterized by inflammation of the gastrointestinal tract. The pathogenesis of IBD involves genetic susceptibility, dysbiosis of the gut microflora, and chronic inflammation triggered by environmental factors such as diet [85]. The Mediterranean Diet, which is rich in certain micronutrients, has shown potential in modulating gut inflammation and could be beneficial in managing IBD. However, specific micronutrients have shown limited benefits in clinical trials, and further research is needed to evaluate the role of individual food compounds in preventing and managing IBD [86].

Animal model studies have shown that LAB are useful in the prevention and treatment of gastrointestinal inflammatory disorders such as IBD and mucositis, indicating their suitability for examination in clinical trials (Table 3) [87]. Research conducted to date has focused on using wild-type or genetically engineered LAB strains that express anti-inflammatory proteins as a promising strategy for treating these gastrointestinal conditions [32]. The mechanism of action by which LAB help prevent mucositis involves a series of interacting biological events that affect all cells and tissues of the mucous membrane, including endothelial apoptosis as the primary initiator of radiation-induced mucosal injury [88]. The specific bacteria involved in preventing mucositis have not been identified. Several studies have examined the administration of antimicrobial agents such as bacteriocins (e.g., nisin, reuterin) as a means of preventing mucositis by reducing levels of factors that stimulate bacterial growth in the oropharynx [89, 90]. However, the LAB does not directly prevent mucositis. Mucositis is a complex biological process resulting from cytotoxic cancer therapy and not affected significantly by changes in saliva or the microflora [91]. Bacteria interact with mucosal cells by adhering to mucus oligosaccharides and then forming microcolonies and biofilms, in which they release and then metabolize monosaccharides from mucin *O*-glycans [92]. Mucus components can affect the behavior of pathogenic bacteria by modulating virulence factor expression, adhesion, motility, and proliferation. Different microbes exploit various components of the mucosal defense system to assist their interactions with mucosal cells [93]. In 2020, Namai et al. proposed that NZ-IL1Ra could be used as an inexpensive and effective tool for the treatment of colitis by targeting IL-1 signaling in the colon; they suggested that the use of NZ-IL1Ra will open the door for broader application of microbial therapeutics [94]. Another study showed that the use of gmLAB to administer a recombinant anti–IL-4 scFv inhibits IL-4 signaling in macrophages. This could be an attractive approach for treating various allergic disorders [95].

**Table 3:** Animal model studies demonstrating the therapeutic potential of LAB for treating gastrointestinal inflammatory disorders

LAB strain/intervention	Route	Key outcomes	Refs
*L. casei BL23*	Oral	Reduced weight loss, diarrhea, colon inflammation; DltD and RecA mutants lost benefits	[186]
*L. casei BL23* (MnKat + catalase)	Oral	Reduced colitis via ROS detoxification; enhanced effect from catalase	[187]
*L. casei Shirota* (LcS)	Oral	Improved body weight, colon length, tight junctions, and epithelial regeneration	[188, 189]
*L. paracasei* R3	Oral	Ameliorated colitis; balanced Th17/Treg cells; reduced inflammation	[190]
*L. rhamnosus* GG (LGG) & EVs	Oral	Live LGG induced IL-10 via STING; EVs suppressed TNF-α, IL-6, IL-1β	[191]
*L. rhamnosus* CY12	Oral	Restored barrier proteins; inhibited TLR4/MyD88/NF-κB	[192, 193]
*L. casei* ATCC 393 + VIP	Oral	Improved DAI, reduced pro-inflammatory cytokines with VIP enhancement	[194]
*L. lactis* NZ9000 secreting IL-1Ra	Oral	Lowered weight loss, DAI, CD4⁺ IL-17A⁺ cells	[94]
*L. lactis* delivering anti-TNFα scFv	Oral	Improved histology; lowered DAI; balanced cytokine levels	[195]
*L. lactis* delivering GDF11	Oral	Alleviated mucosal damage; reduced pro-inflammatory cytokines	[196]
*L. lactis* delivering hCAP18	Oral	Promoted IL-17A, IL-10; alleviated symptoms	[197]
*L. rhamnosus* 2016SWU (Lr-0601)	Oral	Improved barrier function and reduced inflammation	[198]
*L. casei* + phlorotannins (synbiotics)	Oral	Reduced colon shortening and splenic hypertrophy; improved gut barrier	(199)

## Enhancing Mucosal Immunity Against Sexually Transmitted Infections (STIs)

gmLAB have shown promise as mucosal vaccine vectors for the specific treatment of STIs due to their capacity to colonize mucosal surfaces and stimulate robust local immune responses [96]. For instance, *Lactobacillus jensenii* genetically modified to secrete the anti-HIV protein cyanovirin-N provided protection against simian-human immunodeficiency virus transmission in a macaque model, highlighting the potential efficacy of this gmLAB therapy for preventing HIV infection [97]. Recombinant *Lactobacillus plantarum* and *Lactobacillus casei* strains engineered to express HPV antigens were shown to elicit significant mucosal and systemic antibody responses, suggesting these strains would be useful for prophylactic mucosal vaccination as a means of preventing HPV-associated cervical cancer [98, 99]. Similarly, recombinant *Lactobacillus plantarum* strains expressing *Chlamydia trachomatis* major outer membrane protein induced strong protective immune responses in murine models, representing an attractive approach for combating chlamydial infections via mucosal immunization [100].

The female reproductive tract is protected by multiple levels of innate immunity, including the mucus lining and antimicrobial peptides, as well as adaptive immune responses regulated by sex hormones. STIs such as HIV predominantly affect women in various regions, and their immunity is affected by hormonal changes during the menstrual cycle [101]. Vaginal lactobacilli play a role in protecting against viral STIs such as HPV, HIV, and herpes simplex virus (HSV) by maintaining an acidic pH and modulating the immune response [102]. Vaginal pH has a significant impact on the activity of lactobacilli, as the production of lactic acid in the vagina by these bacteria is limited primarily by their sensitivity to elevated hydrogen ion concentration (low pH) rather than lactate concentration [103]. The pH limitation of the lactobacilli predominant among the vaginal microbiota is a critical factor in determining the concentration of antimicrobial lactic acid present. The relationship between vaginal pH and activity of lactobacilli is crucial for maintaining a healthy vaginal microbiome [104]. The average vaginal pH in women with a predominantly lactobacillus-morphotype microbiota is 3.80 ± 0.20, which is tightly correlated with a lactate concentration of 0.79 ± 0.22% (w/v).

## How Probiotic Microorganisms Modulate the Immune System

Probiotic bacteria exhibit a variety of immunotherapeutic effects that strengthen the immune system to fight infections. These effects are attributed to the immunogenic and immune-modulating properties of these bacteria, which also exhibit tumor-reducing activities [105, 106]. The impact of probiotic bacteria on the host immune system is species and strain specific, with various strains eliciting unique mucosal cytokine profiles. Probiotic microorganisms are ingested orally in order to elicit a systemic immune response [107].

The immunomodulatory and vaccine properties of gmLAB primarily depend on the proteins or antigens they express. Yet, it is crucial to ensure these engineered strains retain their inherent probiotic properties after genetic modification [108]. Studies indicate that gmLAB strains such as recombinant *Lactococcus lactis* and *Lactobacillus casei* typically retain their probiotic characteristics, including the ability to survive gastrointestinal passage, adhere to mucosal surfaces, and stimulate beneficial immune responses comparable to their wild-type counterparts [109]. For instance, genetically engineered *L. lactis* strains expressing therapeutic proteins such as IL-10 retain their probiotic capacity to reduce inflammation and promote mucosal homeostasis in IBD models, demonstrating the post-modification preservation of probiotic function [110]. gmLAB also interacts with mucosal and immune cells like probiotic strains through PRRs on the surface of epithelial and immune cells, thereby initiating mucosal immune responses [111, 112]. However, genetic modifications that alter the surface expression or secretion of antigens can enhance their uptake by mucosal-associated APCs or DCs [58].

Various types of immune cells, stromal cells, endothelial cells, and fibroblasts produce cytokines that regulate immune responses. Probiotic bacteria induce the gut mucosal immune response through interactions with gut epithelial cells in order to trigger a cascade of signals that promote an immune response [113, 114]. Nonspecific immune responses involve an inflammatory response mediated by phagocytic cells and macrophages. Bacteria arriving in the colon are absorbed by M cells or the lamina propria of the small intestine. The probiotic bacteria are delivered to and phagocytosed by APCs, macrophages, DCs, and B and T lymphocytes [115]. The defense mechanisms are coordinated by the release of cytokines such as IL-10, IL-6, and IL-2 by mucosal immune epithelial cells, thereby regulating both specific and nonspecific immune responses. The release of cytokines by macrophages and T cells can be stimulated by probiotics [116].

The mucosal immune response is characterized by high levels of secretory IgA, which is triggered by certain probiotic bacteria. This enhances both mucosal and systemic immunity, resulting in the generation of antigen-specific antibodies that target infectious agents [117]. The immune system can be boosted by LAB that stimulates the synthesis of type 1 interferons, which are vital for responses against viruses. The immunological response to self and external antigens can also be impacted by certain LAB strains that induce partial maturation of DCs [118]. Mucus, primarily composed of mucins secreted by gland cells of the intestinal epithelium, is affected by probiotic bacteria that regulate mucin expression, thereby impacting gut immunity [119]. These probiotics promote the growth, adhesion, and translocation of beneficial bacteria while preventing the adhesion of pathogenic strains of organisms such as *Escherichia coli* to the mucus layer [120].

## Strategies for Developing LAB Vaccines

Recent advancements in the development of gmLAB as mucosal vaccine vectors have led to sophisticated strategies to enhance antigen delivery and immunogenicity. These strategies encompass the use of engineered strains for antigen expression, incorporation of immunomodulatory molecules, surface display technologies, and delivery route optimization [121]. One approach involves the co-expression of antigens with immune-stimulating adjuvants. For example, a recombinant *Lactococcus lactis* strain expressing glycoprotein D of HSV-1 along with IL-2 fused to the Fc fragment of IgG demonstrated significant immunogenicity in murine models. Oral administration of this construction elicited both systemic and mucosal responses, characterized by increased serum IgG, fecal IgA, and cytokine levels of IFN-γ and IL-4. Furthermore, this formulation reduced HSV-1–induced lung pathology, illustrating the potential use of LAB co-expressing cytokine-fused antigens to induce robust protective immunity [122].

The use of LAB for multivalent vaccine delivery has also shown promising results. A multicomponent vaccine strategy targeting African swine fever virus (ASFV) employed *L. lactis* strains expressing eight different ASFV antigens (e.g., F317L and p72). In a murine model, oral administration of the cocktail induced antigen-specific IFN-γ and IL-10 responses, whereas intramuscular injection produced higher serum IgG titers. Although fecal sIgA production was transient, the results highlighted the feasibility of using gmLAB for the delivery of complex, multigenic antigens against difficult-to-treat viral pathogens [123].

Surface display systems have also been explored as a means of enhancing antigen presentation. Oral administration in mice of *Lactiplantibacillus plantarum* expressing spike (S1) fragments of SARS-CoV-2 on the cell surface resulted in significant serum IgG production and mucosal IgA responses by day 21 post-immunization. Vaccination also modulated systemic cytokine expression, including that of IFN-γ, IL-4, TNF-α, and IL-10, indicating that vaccination resulted in a balanced Th1/Th2 response essential for antiviral immunity [124, 125].

In addition to surface display, the secretion of antigens into the gut lumen has been employed to enhance mucosal interaction. Oral administration of *L. lactis* secreting *Brucella* lumazine synthase (BLS) utilizing the Usp45 signal peptide induced a mixed Th1/Th2 response, including elevated serum IgG1/IgG2a expression and increased levels of IFN-γ, TNF-α, IL-4, and IL-10. Mice immunized with this construct exhibited reduced splenic inflammation and histological damage upon challenge [126]. Similarly, *L. lactis* expressing *Brucella* Omp10 provided significant protection against infection in a murine model, marked by reduced bacterial load and tissue pathology, further confirming the utility of secreted antigens in LAB vaccines [127].

Another innovative strategy involves co-expression of endogenous bacterial adjuvants such as cyclic di-adenosine monophosphate (c-di-AMP). Co-delivery of antigen-expressing *L. lactis* strains with separate strains expressing c-di-AMP significantly enhanced mucosal and systemic immune responses. In delayed-type hypersensitivity assays, mice immunized with both strains exhibited greater antigen-specific responses than those receiving the antigen alone, suggesting that adjuvant-expressing LAB strains can function synergistically to amplify vaccine efficacy [128, 129].

Typical dosing regimens used in preclinical studies involve oral administration of 10^8^ to 10^9^ colony-forming units (CFU) of recombinant LAB per dose, with schedules varying from single priming doses to multi-dose regimens over 2 to 3 weeks. In the BLS vaccine model, daily oral administration for 2 weeks resulted in significantly greater antibody titers and cellular cytokine responses, demonstrating that repeated low-dose exposure effectively primes mucosal immunity [126, 127].

Mucosal immune responses to bacterial antigens, including those expressed by *Bacillus anthracis*, have been the subject of recent research exploring their potential. The protective antigen (PA) of *B. anthracis* was engineered in *L. acidophilus* to be fused to a 12–amino acid peptide (DCpep) that is bound explicitly to DCs, promotes endocytosis, and is then secreted [130]. The efficacy of this approach was evaluated by oral immunization of mice and subsequent challenge with a lethal dose of the *B. anthracis* Sterne strain. The current recombinant PA vaccine is not optimal, as it is reactogenic in certain individuals and requires multiple subcutaneous administrations.

An additional targeting strategy utilizing the *E. coli* heat labile toxin B (LTB) subunit protein was assessed as a method for enhancing the immune response to *L. casei* expressing a recombinant porcine rotavirus antigen, VP4 [131]. The ADP-ribosylation activity of the A subunit and the GM1-gangliside–binding activity of the pentameric B subunit were demonstrated as rendering the LTB a mucosal adjuvant. Nevertheless, these holotoxins are too toxic for use in humans [132].

The potential for using bacterial flagellin as a fusion partner for vaccine antigens expressed by *L. casei* was also investigated. Flagellins can function as protective antigens per se; however, they can also function as adjuvants when expressed as fusion proteins with other antigens. The SipC antigen of *Salmonella enterica* was expressed by *L. casei* alone and as a fusion protein coupled to the N- or C-terminus of flagellin in the pLP401 vector. Each strain elicited an equivalent response to SipC following intraperitoneal injection, suggesting that flagellin did not function as an adjuvant to enhance the antibody response to this antigen [131].

Several studies have attempted to modulate LAB vaccine responses via the co-expression of cytokines, following the initial demonstration that *L. lactis* can secrete biologically active IL-2 and IL-6 and stimulate mucosal and systemic reactions to the model antigen TTFC. In a mouse cancer model, protection was enhanced by the co-administration of an IL-12–secreting strain of *L. lactis* with another strain of *L. lactis* expressing a cell wall–anchored form of the E7 antigen from HPV-16 [133]. The adjuvant effect of *L. casei* secreting murine IL-1β was recently examined in conjunction with a heat-killed *Salmonella enterica* serovar Enteritidis vaccine [134]. The potential adjuvant effect of Mig-IP-10 was investigated using intranasal administration of an *L. lactis* strain that secretes a Mig-IP-10 fusion protein and cell wall–anchored form of the E7 antigen from HPV or *L. lactis* expressing E7 [135].

## Summary of the last 10 Years of Research Progress on LAB Vaccines

Over the past 10 years, gmLAB have evolved as versatile and effective mucosal vaccine platforms, with significant advancements addressing antigen design, vector engineering, immune modulation, and clinical translation [136]. Researchers have refined gene expression systems in gmLAB, notably *Lactococcus lactis* and *Lactobacillus* spp., by optimizing inducible promoters such as the well-established nisin-controlled expression (NICE) system, enabling high-yield, tightly regulated antigen production with minimal metabolic burden [137, 138]. Constitutive and carbohydrate-inducible promoters have also been exploited to enable in vivo controlled expression responsive to host dietary components [139, 140]. Codon optimization has enhanced heterologous antigen production, as seen with viral proteins such as HPV-16 E6 and rotavirus VP8, improving translational efficiency and immunogenicity in the bacterial host [81, 141]. Additionally, chromosomal integration strategies have advanced stability and regulatory compliance, reducing plasmid loss and antibiotic marker concerns, which is critical for clinical applications [40].

Surface anchoring and extracellular secretion of antigens play pivotal roles in enhancing immune detection and stimulation [142]. Systems utilizing signal peptides such as Usp45 and anchoring motifs such as SlpA have facilitated the presentation of bacterial, viral, and parasitic antigens on the surface of LAB cells, eliciting potent mucosal IgA and systemic IgG responses that are critical for protective immunity [143]. Multivalent vaccines that display several antigens simultaneously have also been developed, demonstrating that co-expression strategies can broaden immune coverage, as exemplified in *Lactococcus lactis* vaccines targeting ASFV [144].

Beyond antigen delivery, gmLAB have been engineered to co-express immune modulators and thereby potentiate vaccine efficacy. Cytokines such as IL-12, IL-2, and IL-4, delivered by recombinant LAB, have successfully skewed immune responses toward desired Th1 or balanced Th1/Th2 profiles, enhancing cellular immunity and antibody production [98, 145]. Endogenous synthesis of c-di-AMP, a known mucosal adjuvant, by gmLAB has emerged as a novel mechanism to stimulate innate immune pathways, augmenting DC activation and T-cell priming [146]. Most preclinical studies employ oral or intranasal delivery routes to harness mucosal immune responses at pathogen entry points [74]. Oral administration targets gut-associated lymphoid tissues, inducing secretory IgA and systemic immunity, whereas intranasal administration promotes respiratory mucosal protection [147]. Dose optimization studies have revealed that repeated administration of 10^8^–10^9^ CFU of recombinant LAB over 2 to 3 weeks optimizes immunogenicity without compromising safety [148]. Preclinical models encompassing bacterial, viral, and parasitic infections have consistently demonstrated the protective efficacy of gmLAB vaccines. For example, administration of *L. lactis* secreting BLS was shown to induce robust Th1/Th2 responses and confer protection against murine brucellosis [148]. Similarly, administration of recombinant *Lactiplantibacillus plantarum* expressing SARS-CoV-2 spike protein induced balanced mucosal and systemic immunity, illustrating the potential for rapid adaptability to emerging pathogens [124]. Clinically, a vaccine employing *L. lactis* that secretes IL-10 has undergone phase I trials for protecting against IBD demonstrated safety and mucosal immunomodulation that supports future development of the vaccine [149].

The integration of synthetic biology has led to programmable LAB vaccine platforms with inducible and environmentally responsive gene circuits, enabling fine-tuned antigen expression and delivery [77]. Combination vaccines employing multiple antigens and co-delivered adjuvants via gmLAB are being developed to broaden protective efficacy for a diverse array of pathogens [128]. Such innovations, coupled with an improved understanding of mucosal immunology and microbiome interactions, position gmLAB at the forefront of next-generation mucosal vaccine technology.

## Limitations and Risks of Vaccination Using Live gmLAB

Vaccines using live gmLAB have gained attention over the last decade for their safety and efficacy. Although these vaccines exhibit several pre-requisite characteristics that make them favorable carriers [150], many questions remain to be answered before they can be marketed. The genes that encode target antigens are either located on the chromosome or a plasmid. It is thus necessary to assess potential risks, including the fate of the recombinant plasmid. In addition to determining which cells take up and express the DNA, studies should also determine what happens to the DNA once it reaches the cell and how much plasmid ends up outside the target cells [151]. Oncogenesis, which involves the genetic and cellular transformation of benign cells into cancerous cells, can be induced by recombinant plasmids present in bacterial vaccines [152]. To avoid undesired plasmid DNA integration, essential sequences can be deleted. Furthermore, some peptides may induce allergic reactions, so careful evaluation is needed before LAB expressing such peptides are used as vaccine antigen carriers [150]. Autoimmunity is another risk associated with live bacterial vaccination, but no convincing data exist regarding the link between vaccination and human autoimmunity. Vaccination involving an adjuvant that activates regulatory T cells could potentially prevent the development of autoimmune diseases [153]. Another concern is the ability of the bacteria to survive in nature, which can be avoided by using auxotrophic mutants that cannot replicate in the environment [154].

## Challenges and Future Directions

gmLAB hold great promise for applications in vaccine development, but several challenges must be addressed to ensure their effectiveness, safety, and scalability. Key challenges include achieving efficient antigen expression, understanding how LAB interacts with the immune system, and ensuring efficient delivery. Advanced genetic engineering techniques, adjuvant properties, and personalized gmLAB could enhance antigen stability and targeted delivery. Rational design strategies, involving careful selection and optimization of bacterial strains, promoters, secretion signals, and antigen presentation systems, play a critical role in enhancing the adjuvant properties, antigen stability, targeted delivery, safety, and overall efficacy of gmLAB vaccines [112, 155]. For example, employing inducible promoters and codon optimization improves controlled antigen expression and protein stability, whereas fusion to mucosal-targeting peptides or co-expression of immunomodulatory molecules such as cytokines enhances mucosal uptake and immune stimulation [156, 157].

Regulatory hurdles in approving mucosal vaccines include safety concerns, efficacy, and manufacturing standards. Future directions include developing standardized protocols for evaluating vaccine safety and efficacy, collaborating with regulatory agencies, and establishing models for successful mucosal vaccine approval. Several challenges could affect the commercialization and widespread use of gmLAB vaccines, such as economic viability, market acceptance, and intellectual property and licensing restrictions. Future directions include the formation of partnerships between academia, industry, and governments, focusing on diseases with significant global health impacts, and developing open-source platforms for LAB-based vaccine design.

## Conclusions

gmLAB represent a groundbreaking platform in mucosal vaccine development that has the potential to transform global immunization practices by enhancing both mucosal and systemic immunity. Although gmLAB vaccines offer promising advantages—including safety, cost-effectiveness, and robust mucosal immune stimulation—they still face significant challenges. These include optimizing antigen expression, navigating complex regulatory approval processes, and developing scalable manufacturing methods necessary for widespread clinical application. Unique to gmLAB is their ability to combine probiotic colonization with targeted antigen delivery at mucosal surfaces, which enhances localized immune responses without provoking inflammation. Their GRAS status and food-grade classification facilitate regulatory acceptance and public trust. Additionally, gmLAB can be produced and stored economically without the need for cold chain logistics, making them especially suitable for deployment in resource-limited settings. Despite these advantages, overcoming the remaining hurdles—such as improving antigen expression, addressing regulatory barriers, and scaling up production—will be essential for the full realization of the potential of gmLAB vaccines. Continued research to better understand the mechanisms of immune response induction by LAB vaccines, alongside the development of innovative genetic engineering techniques and commercialization strategies, will be critical in addressing these challenges. Investing in the development of gmLAB vaccines represents a forward-looking commitment to accessible and effective healthcare. By leveraging the unique capabilities of this promising vaccine vector, we can pave the way toward safer, more affordable, and widely accessible vaccines, particularly benefiting populations in resource-limited areas.

## Data Availability

No datasets were generated or analysed during the current study.
